# Assessing Uncertainty and Reliability of Connective Field Estimations From Resting State fMRI Activity at 3T

**DOI:** 10.3389/fnins.2021.625309

**Published:** 2021-02-22

**Authors:** Azzurra Invernizzi, Nicolas Gravel, Koen V. Haak, Remco J. Renken, Frans W. Cornelissen

**Affiliations:** ^1^Laboratory for Experimental Ophthalmology, University of Groningen, University Medical Center Groningen, Groningen, Netherlands; ^2^Cognitive Neuroscience Center, Department of Biomedical Sciences of Cells and Systems, University Medical Center Groningen, Groningen, Netherlands; ^3^Neural Dynamics of Visual Cognition Group, Department of Education and Psychology, Freie Universität Berlin, Berlin, Germany; ^4^Donders Institute for Brain, Cognition and Behaviour, Radboud University Medical Center, Nijmegen, Netherlands

**Keywords:** resting-state fMRI, uncertainty, connective field modeling, bayesian modeling, visual field mapping, visual cortex

## Abstract

Connective Field (CF) modeling estimates the local spatial integration between signals in distinct cortical visual field areas. As we have shown previously using 7T data, CF can reveal the visuotopic organization of visual cortical areas even when applied to BOLD activity recorded in the absence of external stimulation. This indicates that CF modeling can be used to evaluate cortical processing in participants in which the visual input may be compromised. Furthermore, by using Bayesian CF modeling it is possible to estimate the co-variability of the parameter estimates and therefore, apply CF modeling to single cases. However, no previous studies evaluated the (Bayesian) CF model using 3T resting-state fMRI data. This is important since 3T scanners are much more abundant and more often used in clinical research compared to 7T scanners. Therefore in this study, we investigate whether it is possible to obtain meaningful CF estimates from 3T resting state (RS) fMRI data. To do so, we applied the standard and Bayesian CF modeling approaches on two RS scans, which were separated by the acquisition of visual field mapping data in 12 healthy participants. Our results show good agreement between RS- and visual field (VF)- based maps using either the standard or Bayesian CF approach. In addition to quantify the uncertainty associated with each estimate in both RS and VF data, we applied our Bayesian CF framework to provide the underlying marginal distribution of the CF parameters. Finally, we show how an additional CF parameter, *beta*, can be used as a data-driven threshold on the RS data to further improve CF estimates. We conclude that Bayesian CF modeling can characterize local functional connectivity between visual cortical areas from RS data at 3T. Moreover, observations obtained using 3T scanners were qualitatively similar to those reported for 7T. In particular, we expect the ability to assess parameter uncertainty in individual participants will be important for future clinical studies.

## Highlights

- Local functional connectivity between visual cortical areas can be estimated from RS-fMRI data at 3T using both standard CF and Bayesian CF modeling.
- 3T observations were qualitatively similar to those previously reported at 7T.
- Bayesian CF modeling quantifies the model uncertainty associated with each CF parameter on RS and VF data, important in particular for future studies on clinical populations.

## Introduction

Spontaneous blood-oxygen level dependent (BOLD) fluctuations have been used to study the intrinsic functional connectivity of the human brain. Biswal et al. (1995) observed, for the first time, the presence of bilateral spatial integration, coherent activity and functional connectivity between distant homotopic brain areas, even in the absence of a task. Ever since, resting-state fMRI (RS-fMRI or RS) has played a key role in understanding the temporal and spatial interactions of interconnected brain regions. In parallel, various fMRI data-analysis tools have been developed with the aim to describe the functional and neuroanatomical organization of the brain. One of these methods is connective field (CF) modeling (Haak et al., 2013b). CF, also known as the cortico-cortical population receptive field (cc-pRF), modeling allows to describe the response of a population of neurons in the cortex in terms of the activity in another region of the cortex. It translates the concept of the receptive field into the domain of connectivity by assessing the spatial dependency between signals in distinct cortical visual field regions (Haak et al., 2013b). Even though the approach is agnostic to different stimulus configurations, it has thus far been primarily developed and applied in vision research. A previous study by Gravel et al. (2014) showed that CFs, estimated from RS-fMRI data recorded at a high magnetic field (7T), reflect the visuotopic organization of early visual cortical maps. This indicates that even in the absence of any visual stimulation, CF modeling is able to describe the activity of voxels in a target region (e.g., V2 or V3) as a function of the aggregate activity in a source cortical visual area (e.g., V1).

While these previous results were obtained at 7T and in healthy participants, 3T scanners are much more common, and generally preferred for whole-brain analyses in patient studies (van der Kolk et al., 2013; Polimeni and Uludağ, 2018). Therefore, if RS data recorded at 3T can provide sufficient sensitivity to estimate the spatial integration and connectivity of BOLD signals in distinct regions of the early visual cortex (Gravel et al., 2020), this would open up the CF modeling approach to clinical studies performed at 3T and in individual cases. Amongst others, this would provide the important advantage that plasticity of visual cortical areas could be studied without a dependence on actual visual stimulation. This is important, as in ophthalmic and neurological patients visual input can already be disrupted, potentially resulting in spurious plasticity estimates (Baseler et al., 1999; Azzopardi and Cowey, 2001; Haak et al., 2013b; Carvalho et al., 2020).

In order to assess the suitability of the CF approach for studying unique patient cases at 3T, we will look beyond the classical variance explained as an indicator of modeling performance. To do so, we will assess the uncertainty of model parameter estimates using a Bayesian approach. These parameters are available to us by applying our recently developed Bayesian framework for the CF model (Bayesian CF, Invernizzi et al., 2020). In particular, this approach allows to estimate the variability for each CF parameter estimate such as CF size and beta. Moreover, when using our new Bayesian CF framework, we can obtain a data-driven threshold in order to select relevant voxels for both RS-fMRI and visual field mapping (VFM) data.

We applied both the standard CF estimation and the novel Bayesian approach to RS and VFM data acquired at 3T. Subsequently, we compared the CF maps and parameters obtained using the two CF approaches. Additionally, we assessed test-retest reliability between the two runs of RS data.

Finally, we will qualitatively compare our results to those obtained previously in Gravel et al. (2014).

To preview our results, we found a good agreement between RS- and visual field (VF) – based maps obtained with both the standard and Bayesian CF approach at 3T. Moreover, most observations were qualitatively similar to those previously observed for 7T data. This implies that local functional connectivity between visual cortical areas during RS can be estimated at 3T. No significant differences were found between the two runs of RS data on V1 > V2 areas. Furthermore, we showed how the parameter uncertainty can be used to assess the variability of parameters in RS-fMRI BOLD fluctuations. Therefore, the Bayesian CF approach presented here extends on previous approaches to provide an interpretable and independent measure of uncertainty in RS-based data. Finally, we show that the novel retained CF parameter, *beta*, can serve as a sensitive threshold for the selection of voxels and improve the reliability of estimates. Taken together, our results demonstrate the utility of applying a Bayesian CF approach to study inter areal cortical integration in the human visual cortex in health and disease.

## Materials and Methods

### Participants

Twelve healthy female participants (mean age 22 years, s.d. = 1.8 years) with normal or corrected-to-normal vision and without a history of neurological disease were included. These data had been collected and used in previous projects (Halbertsma et al., 2019; Invernizzi et al., 2020). For each of the previous studies, the ethics board of the University Medical Center Groningen (UMCG) approved the study protocol. All participants provided written informed consent. The study followed the tenets of the Declaration of Helsinki.

### Stimuli Presentation and Description

The visual stimuli were displayed on a MR compatible screen located at the head-end of the MRI scanner with a viewing distance of 118 cm. The participant viewed the complete screen through a mirror placed at 11 cm from the eyes supported by the 32-channel SENSE head coil. Screen size was 36 × 23° of visual angle and the distance from the participant’s eyes to the screen was approximately 75 cm. Stimuli were generated and displayed using the Psychtoolbox^1^ and VISTADISP toolbox (VISTA Lab, Stanford University), both Matlab based (Brainard, 1997; Pelli, 1997). The stimulus consisted of drifting bar apertures (of 10.2° radius) with a high contrast checkerboard texture on a gray (mean luminance) background. A sequence of eight different bar apertures with four different bar orientations (horizontal, vertical, and diagonal orientations), two opposite motion directions and four periods of mean-luminance presentations completed the stimulus presentation that lasted 192 s. To maintain stable fixation, participants were instructed to focus on a small colored dot present in the center of the screen and press a button as soon as the dot changed color. The complete visual field mapping paradigm was presented to the participant six times, during six separate scans.

### Resting State

During the RS-fMRI scans, the stimuli were replaced by a black monitor and the lights in the scanning room were turned off. All participants were instructed to keep their eyes closed, remain as still as possible, not to fall asleep, and try not to think of anything in particular.

### Data Acquisition

Magnetic resonance imaging (MRI) and fMRI data were obtained using a 3T Philips Intera MRI scanner (Philips, Netherlands), with a 32-channel head coil. For each subject, a high-resolution T1-weighted three-dimensional structural scan was acquired (TR = 9.00ms, TE = 3.5 ms, flip-angle = 8, acquisition matrix = 251 mm × 251 mm × 170 mm, field of view = 256 × 170 × 232, voxel size = 1 mm × 1 mm × 1 mm). Then, six retinotopy (VFM) functional T2^∗^-weighted, 2D echo planar images were obtained (TR = 1500 ms, field of view = 224 mm × 72 mm × 193.5 mm, voxel resolution of 2.33 mm × 2.33 mm × 3 mm). Finally two, full brain, resting-state (RS) functional T2^∗^-weighted, 2D echo planar images were obtained using the following parameters: TR = 2000 ms, field of view = 220 mm × 121 mm × 220 mm, voxel size = 3.44 mm × 3.44 mm × 3.29 mm. The functional scans were acquired in the following order: (1) a RS-fMRI scan (RS1) lasted 708 s with a total of 350 volumes; (2) six VFM functional scans were collected, where each scan lasted for 204 s with a total of 136 volumes; (3) finally, a second RS-fMRI scan (RS2) with the same characteristic of RS1 (duration of 708 s with 350 volumes) was collected. MRI protocol differences between VFM and RS scans are due to the fact that RS was collected at whole-brain while VFM scans were geared to the visual areas in the occipital brain areas.

Prior to the first VFM scan, a short T1-weighted anatomical scan with the same field of view chosen for the functional scans were acquired and used for obtaining a better co-registration between functional and anatomical volume.

### Data Analysis

Preprocessing and standard CF analysis of fMRI data were done using ITKGray^2^, FreeSurfer (Fischl, 2012) and mrVista toolbox for Matlab environment (VISTASOFT)^3^. The Bayesian pRF and CF approaches were developed and implemented in Matlab 2016b (The MathWorks Inc., Natick, MA, United States). The code for the Bayesian pRF and CF frameworks will be made available via www.visualneuroscience.nl.

For each participant, the structural scan was aligned in a common space defined using the anterior commissure-posterior commissure line (AC-PC line) as reference. Next, gray and white matter were automatically segmented using FreeSurfer and manually adjusted using ITKGray^4^, in order to minimize segmentation errors. Then, all functional data were pre-processed using mrVista toolbox. For both RS and retinotopy data the following steps are applied. First, head motion within and between scans were corrected by using robust multiresolution alignment of MRI brain volumes (Nestares and Heeger, 2000) an alignment of functional data into anatomical space and an interpolation of functional data with segmented anatomical gray and white matter. For RS-fMRI data, a few additional denoised steps were applied. These steps were possible since RS scans were acquired at the whole brain. First, spatial smoothing of 6 mm FWHM was applied in order to perform the denoising step based on ICA-AROMA that identified noise and motion related components (Pruim et al., 2015). These components were then removed from the unsmoothed RS-fMRI data that are now further filtered by applying a band-pass filter with high-pass discrete cosine transform with cut-off frequency of 0.01 Hz and a low-pass 4th order Butterworth filter with cut-off frequency of 0.1 Hz.

### Bayesian Population Receptive Field Mapping Applied to VFM

Retinotopy scans were analyzed using a Bayesian population receptive field (pRF) framework. For a detailed account see Prabhakaran et al. (2020), which uses a Markov Chain Monte Carlo (MCMC) approach to sample the parameter space for the pRF mapping. Following the nomenclature of Dumoulin and Wandell (2008), Zeidman et al. (2018), Carvalho et al. (2020), Prabhakaran et al. (2020), we defined 2D symmetrical Gaussian kernel centered at (*x*_0_,*y*_0_) with width defined as the standard deviation σ, to define the pRF model. The best model fit was projected onto a smoothed 3D mesh of the cortex. Based on the obtained parameter-values, visual areas are outlined (V1, V2, V3, hV4, LO1, and LO2) to act as source (V1) or target region (all other) for subsequent RS analysis.

### Standard Connective Field Mapping of RS Data

In the standard CF model, the optimal CF parameters (CF position and CF size, which define the 2D symmetric Gaussian kernel) were estimated based on a procedure that fitted the time-series for each location in the target region (e.g., V2 or V3) using a linear combination of the time-series in the source region (e.g., V1; Haak et al., 2013b). The best fitting models are retained and projected on a smoothed 3D mesh. The CF parameters associated with the best fitting model are converted from cortical units (cortical position) into visual field units (eccentricity and polar angle). This is done by inferring the pRF properties – obtained via the Bayesian pRF method Prabhakaran et al., 2020 of the center voxel in the source region for each target location (Haak et al., 2013b).

### Bayesian Connective Field Mapping

Similar to the Bayesian pRF, the Bayesian CF framework uses a Markov Chain Monte Carlo (MCMC) approach to sample the source region efficiently. Again we used a 2D symmetric Gaussian kernel to predict the time series of the target regions. As in the standard CF modeling, the eccentricity and polar angle values associated with the CF centers are inferred from a pRF mapping. For the sake of completeness, the entire fitting procedure of the Bayesian CF model (option B) is described in the Supplementary Material.

For each participant, standard and Bayesian-CF models were estimated for both VFM and RS data. Target and source regions definitions were based on the Bayesian pRF analysis. For both Bayesian pRF and CF models, a total of 15000 iterations were computed, where the first 10% of iterations were discarded for the burn-in process (Chib, 2011; Liu et al., 2016) and the posterior probability distributions were estimated on the remaining samples.

### Spatial Analysis

We used Pearson and circular correlations to compare and assess the topographic organization of eccentricity and polar angle, respectively, in both standard CF and Bayesian CF maps obtained on RS and VFM data. The same type of correlations were used to evaluate similarities in eccentricity and polar angle maps between the two RS-fMRI scans obtained with standard CF and Bayesian CF models. Furthermore, we computed the coefficient of variation (Shoukri et al., 2008) to evaluate the within-subject reproducibility of CF parameter estimates obtained by using the standard CF and Bayesian CF. Correlation values higher than 0.5 and *p*-values below 0.05 were considered statistically significant. Moreover, to compare the relation between CF size and eccentricity RS-based, we binned the eccentricity at 1° intervals and applied a linear fit over the mean per bin. A confidence interval (CI) of the fit was defined by applying a bootstrap technique 1000 times.

For the spatial analyses, only voxels for which the best-fitting CF model explained more than 15% of the time-series variance in the standard CF and eccentricity which is <1° and >7° were included. This arbitrary threshold level was chosen based on previous literature (Winawer et al., 2010; Baseler et al., 2011; Haak et al., 2012, 2013b).

Finally, the intraclass correlation coefficient (ICC) (McGraw and Wong, 1996; Perinetti, 2018) was computed to estimate the test-retest reliability between the two RS-fMRI scans obtained with standard CF and Bayesian CF models. *A priori* 5% strongest activated voxels based on VE was used as threshold to compute the ICC score. We also report the relation between ICC and five different thresholds (1%, 5%, 10%, 25% and 50%, Supplementary Figure 4).

### Bayesian Analysis

Based on a quantile analysis of the posterior distribution (Invernizzi et al., 2020), we computed a voxel-wise uncertainty measure for each CF parameter by subtracting the upper (*Q*_*3*_) and lower (*Q*_*1*_) quantile of the posterior distribution. The estimated uncertainty was computed for both RS and VFM data for each participant and then projected onto a smoothed 3D mesh of the cortex. We repeated the same procedure for each CF parameter.

### Beta Threshold

Following the procedure reported by Invernizzi et al. (2020) we test if *beta* – the scaling amplitude of the predictor to the amplitude of the measured signal – can serve as data-driven threshold for RS-data. As a proxy distribution for the null hypothesis (i.e., no correlation between source and target region), one surrogate BOLD time series was calculated for each voxel (Schreiber and Schmitz, 1996; Räth and Monetti, 2009; Lancaster et al., 2018). A surrogate time series was generated using the iterative amplitude adjusted Fourier transform method (IAAFT, Schreiber and Schmitz, 1996; Räth and Monetti, 2009). Then, the Bayesian CF model was fitted using this surrogate to real time series of the target region which were unchanged. Based on the best fit obtained in the MCMC iterations of the surrogate *beta*-estimate, we calculated a familywise error (FWE) corrected *beta-*threshold for all the voxels in the target region. Based on previous literature, we selected the cut-off value of the 95th percentile (Bornmann, 2013; Invernizzi et al., 2020) of the null distribution as FWE-corrected *beta* threshold. Finally, we compared the voxel selection at the single participant level using VE and the FWE *beta-*threshold approaches.

## Results

The CF maps obtained from RS-based data for eccentricity, polar angle and CF size were qualitatively comparable for the standard and Bayesian CF models. In contrast to the VFM data, the relation with CF size and eccentricity in RS data does not increase with visual hierarchy. Again, the same behavior was observed using both methods. No statistically significant difference was found between the two RS scans for any CF parameter. We estimated the uncertainty for the Bayesian CF parameters (CF size and beta). An higher uncertainty from the CF parameters was observed from both RS scans compared to VFM data and between RS2 and RS1 scans. Finally, we showed how to use a new threshold based on the effect size of the model both in the presence and absence of visual stimuli.

### CF Models Based on RS-fMRI Data

**FIGURE 1:**
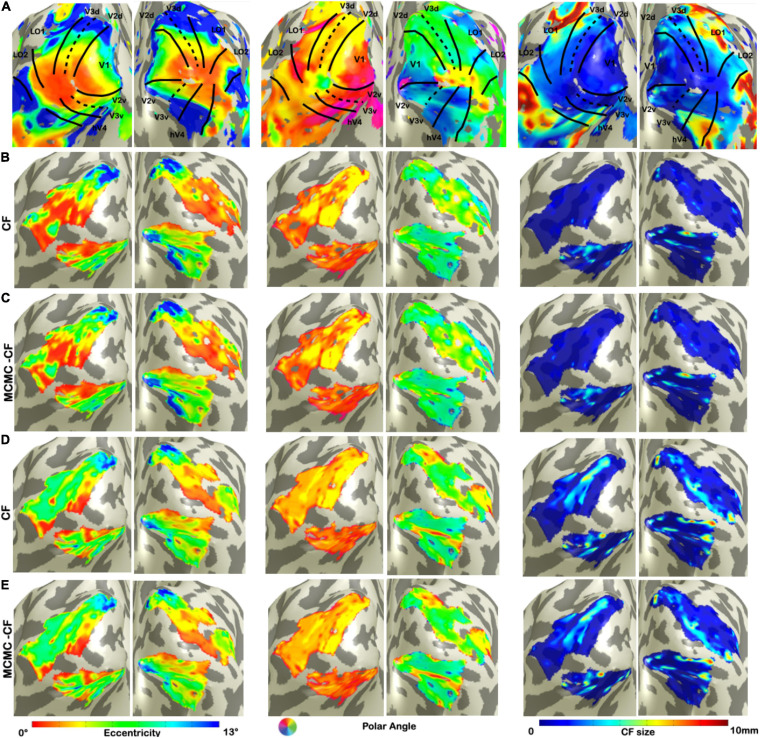
Visualization of CF maps of denoised data for a single participant. From left to right: eccentricity, polar angle, and CF size. **(A)** Corresponds to VFM derived estimates. **(B,C)** Show parameter estimates for the first RS run (RS1) using standard CF and Bayesian CF models, respectively. **(D,E)** Show parameter estimates for the second RS run (RS2) using standard CF and Bayesian CF models, respectively. The fact that **(B,C)** on the one hand, and **(D,E)** on the other, are very comparable is important and indicates that the standard and Bayesian CF models produce highly similar results on RS data.

We used V1 as source region while V2, V3, hV4, LO1, and LO2 as target to derive CF maps projected on a smoothed 3D mesh on a single subject level (Figure 1). Such maps were created using both standard CF and Bayesian CF models (Figures 1B–E). Topographical maps for eccentricity, polar angle and CF size were comparable for both CF models using RS data (Figure 1). We used the VFM-based maps as reference (Figure 1A) as these maps show a clear visuotopic organization for all the CF parameters estimated. Then the same parameters are plotted for all RS scans (Figures 1B,D: RS-based derived maps using standard CF model; Figures 1C, E: RS-based derived maps using Bayesian CF model). Furthermore, a good level of within-subject reproducibility was observed for each CF parameter estimate for both CF models using RS scans (Supplementary Table 2).

In line with the earlier work that introduced the standard CF method (Haak et al., 2013a), we quantified possible differences between the resulting RS-based CF and Bayesian-CF estimates by correlating them against the pRF-derived eccentricity and polar angle parameters that we used as reference (Table 1). Eccentricity and polar angle parameters are estimated for each single participant and then concatenated across participants to calculate the Spearman’s correlation and circular correlation, respectively. Overall a good agreement was found for V1 > V2 and V1 > V3 areas using both CF models. Negative or almost zero correlation values can be observed for CF estimates between distant visual areas (i.e., V1 > LO1, V1 > LO2).

**TABLE 1:** Group level correlation between visual field and resting state maps derived using Bayesian pRF and CF modeling.

**Eccentricity**									
***ROIs***	**Standard CF versus pRF**								
	***VFM***			***RS1***			***RS2***		
	***r***	***IQR [Q1, Q3]***	***p-value***	***r***	***IQR [Q1, Q3]***	***p-value***	***r***	***IQR [Q1, Q3]***	***p-value***

*V1 –> V2*	0.86	[0.82, 0.91]	*p* < 0.001	0.44	[0.38, 0.54]	*p* < 0.001	0.57	[0.47, 0.62]	*p* < 0.001
*V1 –> V3*	0.82	[0.76, 0.87]	*p* < 0.001	0.22	[0.13, 0.34]	*p* < 0.001	0.31	[0.17, 0.43]	*p* < 0.001
*V1 -> hV4*	0.81	[0.73, 0.83]	*p* < 0.001	0.02	[−0.09, 0.40]	0.0048	0.34	[0.04, 0.46]	*p* < 0.001
*V1 -> LO1*	0.78	[0.72, 0.81]	*p* < 0.001	0.07	[−0.13, 0.24]	0.0018	0.15	[0.04, 0.3]	0.0021
*V1 -> LO2*	0.63	[0.43, 0.75]	*p* < 0.001	–0.06	[−0.27, 0.17]	0.0154	0.08	[−0.04, 0.27]	0.0567

***ROIs***	**Bayesian CF versus pRF**								
	***VFM***			***RS1***			***RS2***		
	***r***	***IQR [Q1, Q3]***	***p-value***	***r***	***IQR [Q1, Q3]***	***p-value***	***r***	***IQR [Q1, Q3]***	***p-value***

*V1 –> V2*	0.86	[0.82, 0.91]	*p* < 0.001	0.48	[0.34, 0.57]	*p* < 0.001	0.58	[0.51, 0.63]	*p* < 0.001
*V1 –> V3*	0.82	[0.77, 0.87]	*p* < 0.001	0.25	[0.09, 0.43]	*p* < 0.001	0.3	[0.16, 0.41]	*p* < 0.001
*V1 -> hV4*	0.79	[0.73, 0.84]	*p* < 0.001	0.08	[−0.16, 0.35]	0.0085	0.28	[0.06, 0.40]	*p* < 0.001
*V1 -> LO1*	0.78	[0.72, 0.82]	*p* < 0.001	0	[−0.17, 0.28]	0.0667	0.24	[0.10 0.43]	*p* < 0.001
*V1 -> LO2*	0.65	[0.54, 0.76]	*p* < 0.001	–0.03	[−0.25, 0.18]	0.0641	0.09	[−0.02, 0.38]	0.2264

**Polar Angle**									

***ROIs***	**Standard CF versus pRF**								
	***VFM***			***RS1***			***RS2***		
	***r***	***IQR [Q1, Q3]***	***p-value***	***r***	***IQR [Q1, Q3]***	***p-value***	***r***	***IQR [Q1, Q3]***	***p-value***

*V1 –> V2*	0.92	[0.85, 0.93]	*p* < 0.001	0.64	[0.41, 0.82]	*p* < 0.001	0.78	[0.178, 0.84]	*p* < 0.001
*V1 –> V3*	0.84	[0.78, 0.89]	*p* < 0.001	0.17	[−0.05, 0.82]	0.002	0.26	[−0.53, 0.49]	*p* < 0.001
*V1 -> hV4*	0.79	[0.51, 0.92]	*p* < 0.001	–0.29	[−0.62, 0.66]	*p* < 0.001	0.54	[−0.31, 0.77]	*p* < 0.001
*V1 -> LO1*	0.79	[0.55, 0.89]	*p* < 0.001	0.68	[0.34, 0.76]	*p* < 0.001	0.56	[−0.11, 0.72]	*p* < 0.001
*V1 -> LO2*	0.72	[0.46, 0.76]	*p* < 0.001	0.49	[−0.23, 0.68]	0.0046	0.62	[−0.46, 0.76]	*p* < 0.001

***ROIs***	**Bayesian CF versus pRF**								
	***VFM***			***RS1***			***RS2***		
	***r***	***IQR [Q1, Q3]***	***p-value***	***r***	***IQR [Q1, Q3]***	***p-value***	***r***	***IQR [Q1, Q3]***	***p-value***

*V1 –> V2*	0.92	[0.87, 0.93]	*p* < 0.001	0.62	[0.40, 0.85]	*p* < 0.001	0.83	[0.39, 0.87]	*p* < 0.001
*V1 –> V3*	0.84	[0.70, 0.92]	*p* < 0.001	0.17	[−0.03, 0.68]	*p* < 0.001	0.17	[−0.36, 0.48]	*p* < 0.001
*V1 -> hV4*	0.79	[0.51, 0.92]	*p* < 0.001	–0.34	[−0.55, 0.17]	*p* < 0.001	0.54	[−0.02, 0.71]	*p* < 0.001
*V1 -> LO1*	0.82	[0.55, 0.90]	*p* < 0.001	0.35	[0.10, 0.64]	0.0045	0.52	[−0.08, 0.79]	*p* < 0.001
*V1 -> LO2*	0.67	[0.51, 0.82]	*p* < 0.001	0.24	[−0.22, 0.76]	0.0021	0.3	[−0.35, 0.83]	*p* < 0.001

*Correlations coefficients were computed in order to assess the level of agreement between the eccentricity (Pearson’s correlation) and polar angle (circular correlation) estimates obtained by using standard CF and Bayesian CF models and the ones derived by the pRF. We used the VFM-based pRF estimates as reference as stimuli driven and they further show a clear visuotopic organization for all parameters. Correlation, *p*-values and interquartile range values were estimated at single subject level and then concatenated across all participants.*

To check the possible influence of the denoise procedure applied to RS data, the same quantification analysis was computed on non-denoised RS data. Similar results were observed indicating that the ICA-AROMA denoise procedure on RS-fMRI data did not influence the final CF outcomes. A complete overview of these analyses is reported in Supplementary Material (Supplementary Figure 1 and Supplementary Table 1).

### Test-Retest Reliability

To estimate test-retest reliability between the two RS scans, we selected the 5% most active voxels and computed the ICC score for each parameter estimate obtained using both CF and Bayesian CF model. For completeness, the relation between ICC and chosen threshold is displayed in Supplementary Figure 4. A positive ICC value is reported for V1 > V2 using both models. For higher order visual areas this ICC value gradually drops for all parameters Table 2.

**TABLE 2:** Test-retest evaluation between RS scans.

**Eccentricity**				
***ROIs***	***CF standard***		***Bayesian CF***	
	***ICC (r)***	***IQR [Q1, Q3]***	***ICC (r)***	***IQR [Q1, Q3]***

*V1 –> V2*	0.9661	[0.943, 0.983]	0.9061	[0.844, 0.948]
*V1 –> V3*	0.2072	[0.073,0.462]	0.193	[0.074, 0.313]
*V1 -> hV4*	0.1045	[−0.012, 0.426]	–0.006	[−0.065, 0.503]
*V1 -> LO1*	0.0909	[−0.109, 0.343]	–0.0194	[−0.225, 0.235]
*V1 -> LO2*	0.1036	[−0.184, 0.604]	0.0413	[−0.244, 0.221]

**Polar Angle**				

***ROIs***	***CF standard***		***Bayesian CF***	
	***ICC (r)***	***IQR [Q1, Q3]***	***ICC (r)***	***IQR [Q1, Q3]***

*V1 –> V2*	0.9295	[0.874, 0.976]	0.8717	[0.720, 0.941]
*V1 –> V3*	0.3365	[0.179, 0.495]	0.2997	[0.075, 0.463]
*V1 -> hV4*	–0.027	[−0.081, 0.157]	–0.0583	[−0.209, 0.067]
*V1 -> LO1*	0.0732	[−0.106, 0.536]	0.1944	[−0.053, 0.48]
*V1 -> LO2*	0.4057	[0.137, 0.734]	0.2635	[0.064, 0.6036]

**CF size**				
***ROIs***	***CF standard***		***Bayesian CF***	
	***ICC (r)***	***IQR [Q1, Q3]***	***ICC (r)***	***IQR [Q1, Q3]***

*V1 –> V2*	0.2939	[0.168, 0.396]	0.2192	[0.061, 0.344]
*V1 –> V3*	0.0214	[−0.029, 0.139]	0.0335	[−0.077, 0.201]
*V1 -> hV4*	–0.0533	[−0.131, 0.147]	0.0717	[−0.042, 0.156]
*V1 -> LO1*	–0.0308	[−0.153, 0.049]	–0.0292	[−0.166, 0.076]
*V1 -> LO2*	–0.075	[−0.144, −0.017]	–0.1147	[−0.141, −0.007]

*For eccentricity, polar angle, and CF size estimates obtained by using the standard CF and the Bayesian CF models. Intraclass correlation coefficients (ICC) were computed across all participants and for each ROI separately. Median and interquartile range are computed across the group.*

### Assessing Uncertainty in RS-fMRI Data

**FIGURE 2:**
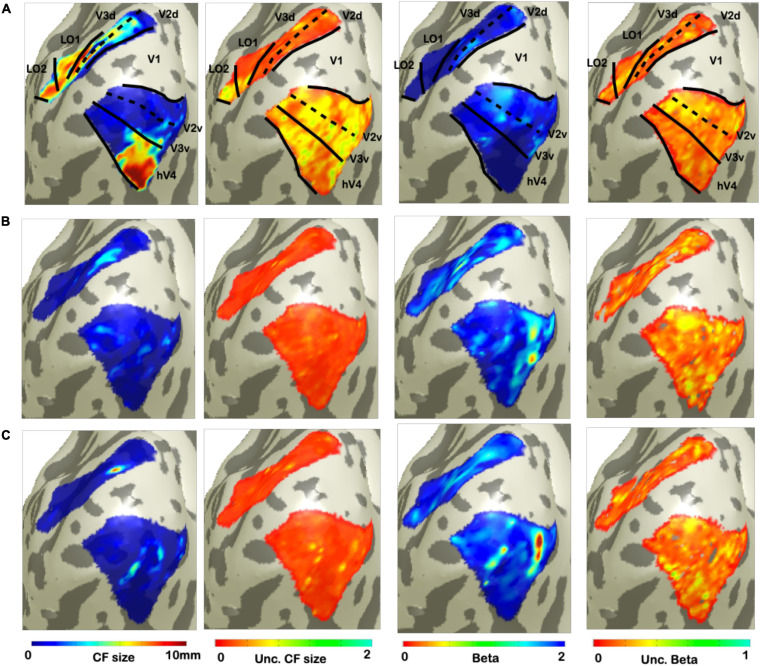
Visualization of uncertainty for CF parameter at single participant. From left to right: CF size, uncertainty of CF size, beta, and uncertainty of beta. **(A)** Corresponds to VFM derived estimates. While, bottom **(B,C)** show the parameters and uncertainty estimates for each of the two RS scans.

In order to estimate a voxel-wise uncertainty value associated to each CF parameter, we computed a quantile analysis of the posterior distribution for each participant. Then, for illustrative purposes, we projected on a smoothed 3D mesh the uncertainty estimates obtained for a single participant (Figure 2), where V1 is the source region and V2, V3, hV4, LO1, and LO2 were the target regions; VFM-based CF maps were used as reference (Figure 2A). An increased uncertainty in beta estimate in RS1- and RS2-based CF maps was observed compared to VFM-based CF maps but not for CF size. Interestingly, no clear uncertainty-related visuotopic organization was found either for VFM or RS data. Furthermore, we evaluated the possible dependency between the Bayesian parameter estimates and the corresponding (posterior) uncertainty by computing the cross-correlation coefficient between these estimates (Table 3). In line with the findings reported in Bornmann (2013), Invernizzi et al. (2020) for VFM data, a weak correlation exists between beta, sigma parameter estimates and their respective uncertainties obtained on RS-data (Table 3). Again, this indicates that uncertainty is an additional, independent parameter, but this time obtained from resting-state data.

**TABLE 3:** Dependency between Bayesian CF parameters and uncertainties for both RS scan at group level.

	**V1** > **V2**		**V1** > **V3**		**V1** > **hV4**		**V1** > **LO1**		**V1** > **LO2**	
**RS1**	***CF size***	***Beta***	***CF size***	***Beta***	***CF size***	***Beta***	***CF size***	***Beta***	***CF size***	***Beta***
*Unc. CF size*	0.06	–0.01	0.11	0.04	0.1	0.07	0.15	0.1	0.1	0.09
*Unc. Beta*	–0.03	0.01	–0.01	0.01	–0.04	0.01	–0.03	–0.12	–0.01	–0.01

**RS2**	***CF size***	***Beta***	***CF size***	***Beta***	***CF size***	***Beta***	***CF size***	***Beta***	***CF size***	***Beta***

*Unc. CF size*	0.08	–0.08	0.06	–0.05	0.04	–0.02	0.03	0.02	–0.02	–0.06
*Unc. Beta*	0.01	–0.01	0.01	–0.02	0.01	–0.03	–0.03	–0.04	–0.02	0.04

*Cross-correlations were computed between the estimated Bayesian CF parameters and the uncertainty derived from them. Only the CF parameters directly estimated using the model (CF size and beta) are included in this analysis. CF size, beta, and their associated uncertainties were estimated at single participant level and then concatenated across all participants.*

### Bayesian CF Thresholding Application

**FIGURE 3:**
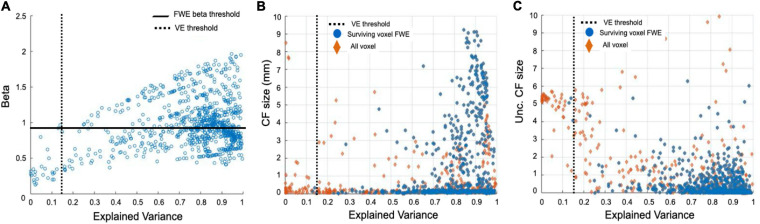
Comparison of thresholding approaches at a single participant level for V1 > V2 connectivity based on RS1 data. In **Panel (A)** the FWE *beta*-corrected thresholds obtained with both 95% CI and the standard VE of the model are shown. A direct comparison between the FWE threshold and the standard VE is presented in **Panels (B,C)**. Since we are interested in testing this FWE-corrected threshold in different conditions, CF size ∼0 which are discarded. In **Panel (B)** the relation between VE and the Bayesian CF size is presented for all voxels (orange diamonds). Blue dots indicate the voxels surviving the 95% CI FWE beta-threshold. The standard VE threshold is not applied but indicated by a black dotted line. In **Panel (C)**, the relation between VE and the uncertainty associated with the CF size is presented. Note that high uncertainty can be associated with voxels with a high VE.

To evaluate the goodness of the corrected beta-thresholding method in the voxel selection on RS data, we compared the model VE, each CF parameter and the uncertainty associated, respectively (Figure 3, CF size and Supplementary Figure 2, beta parameter). Both thresholds: VE is higher than 15% and the FWE corrected effect size (>95% Figure 3A; for more details, see Invernizzi et al. (2020) are indicated. Based on a direct comparison of FWE *beta*-corrected threshold (CI) to the standard VE of the model (Figures 3B,C), the 95% FWE CI-based threshold proved to be more conservative. Note that it is not straightforward to identify a point at which the two threshold definitions will be equivalent.

**FIGURE 4:**
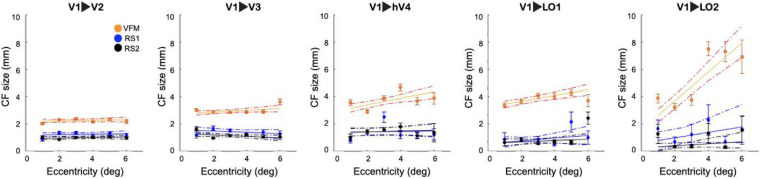
Connective field size as a function of VFM- based eccentricity for RS and VFM scans. For both RS and VFM scans, eccentricity was binned in intervals of 1° and a linear fit was applied. The average CF size was calculated only for voxels that survived the FWE 95%CI threshold. Each dot indicates the mean of the CF size in each bin. The dashed lines correspond to the 95% bootstrap confidence interval of the linear fit. For reference, the VFM data is included.

This threshold was then used to compare the relation between RS-based CF size and VFM-derived eccentricity. Figure 4 shows that RS-based CF size does not increase with eccentricity within the early visual areas. While it is possible to notice an increase of CF size values with eccentricity only for the later visual areas (LO1 and LO2), especially in RS2. However, no significant differences were found between RS1 and RS2 scans in areas along the visual hierarchy.

## Discussion

In this study, we show that 3T RS-fMRI data is suitable for estimating local functional connectivity between visual cortical areas. Furthermore, we observed a good level of agreement between the standard and Bayesian (MCMC) CF models. This indicates that also the latter tool is suitable for studying the cortico-cortical properties of brains at rest. The obtained CF estimates are qualitatively similar to those previously observed for 7T RS-fMRI data. This further supports that sensitive estimations and associated uncertainties can be derived from 3T RS-fMRI data. Finally, we show that a FWE-corrected threshold can be used as a complementary threshold to the standard VE to increase the reliability of estimates. This indicates that both stimulus-driven and RS-based CF modeling are suitable approaches for use in patient- or single-case studies. Below, we discuss our findings in more detail.

### Comparable CF Estimates Based on Resting-State and Visual Field Mapping at 3T fMRI

The CF method was previously used to reveal relevant aspects of resting-state brain activity using high-resolution 7T-fMRI. Crucially, in this study, we have extended the CF approach and assessed its performance at a lower-field strength (3T-fMRI). Higher magnetic fields can increase the signal-to-noise ratio, the tissue specificity and the spatial resolution of fMRI recordings. However, 3T scanners are much more abundant and more often used in clinical research than 7T ones. Our present findings indicate that, despite the limited resolution of metabolism-sensitive measurements such as fMRI for determining the contribution of neuronal activity to hemodynamic signals, it is still possible to study the aggregate neuronal population properties at 3T using CF approaches. A good level of agreement was found between the CF and Bayesian CF maps estimated from RS and those estimated based on VFM for all CF parameters in the early visual areas (Figure 1 and Table 1 – V1 > V2, V1 > V3). Our quantitative and qualitative results are in agreement with those presented previously (Haak et al., 2013b; Gravel et al., 2014; Invernizzi et al., 2020). Qualitatively, we find that the CF maps obtained at 3T are in fair agreement to those obtained at 7T (Gravel et al., 2014, Figure 1). Moreover, while we observed variability in the CF maps estimated for different RS scans, this was also observed previously at 7T Gravel et al. (2014).

Thus, RS-derived CF maps at least partly reflect the functional topographic organization revealed by pRF mapping — regardless of the lower spatial resolution and signal-to-noise ratio afforded by 3T-fMRI. While higher magnetic field strengths allow for an enhanced spatiotemporal resolution, the temporal resolution of fMRI is limited by the hemodynamic response to neuronal activity, not by the magnetic field strength. This suggests that the spatially weighted temporal correlations, as captured by the CF method, suffice to reveal the underlying retinotopically organized connectivity between areas.

Examining the relationship between CF size and pRF eccentricity revealed that RS-derived CF size did not increase with eccentricity, neither within individual areas nor throughout the visual hierarchy (Figures 2, 4). In contrast, for VFM, we did find increased CF sizes at higher pRF eccentricities (Supplementary Figure 3). This was most pronounced for higher-order visual area LO2. The same trend was observed in previous results obtained at 7T (Gravel et al., 2014).

In addition, we investigated whether spatial structure could be observed in the uncertainty information, which could potentially be due to large-scale network interactions, physiological processes or measurement noise. In order to do so, we compared the uncertainties associated with CF size, effect size (beta) in the different conditions (Figure 2 and Table 3). However, neither in the VFM nor in the RS based uncertainty maps did we observe a clear visuotopic organization (Figure 2). Moreover, for the RS data, we find only weak correlations between the CF size and beta and their corresponding uncertainties (correlation <0.25). This is similar to what we observed previously for VFM data (Invernizzi et al., 2020). Therefore, we conclude that, given these rather weak (co)dependencies, the uncertainties can be treated as additional and independent CF parameters describing the RS state data.

### The Effect Size as a New Approach for Voxel-Wise Thresholding of RS Data

The Bayesian variant of CF modeling, in addition to the uncertainties for the CF parameters, provides also a parameter beta describing effect size (β). This parameter can be used to threshold data in a voxel-wise manner similar to the estimated Variance Explained (VE). Since VE indicates the goodness of fit for the model, the current standard approach is to threshold voxels based on the VE of the model for both VFM and RS data (Haak et al., 2013b; Gravel et al., 2014; Halbertsma et al., 2019). However, a high VE does not always correspond to a low uncertainty in the parameter estimates (Thielen et al., 2019). As previously shown for VFM data, beta thresholding provides an alternative thresholding approach (Invernizzi et al., 2020). Here, we show that a FWE-corrected *beta* threshold based on the 95% CI also provides a valid approach for RS data and compares favorably to VE thresholding (Figure 3 and Supplementary Figure 2). However, some thought should be given before applying it to RS data. On the one hand, *beta* thresholding is more sensitive in the selection of voxels compared to the standard VE. Given that RS data is inherently more noisy than VFM data and this might affect the applicability of *beta*-thresholding for this type of scan. On the other, a marked advantage of the FWE *beta*-thresholding approach is that it is participant-specific (Invernizzi et al., 2020). Therefore, using it ensures minimizing the loss of individual participant data and is expected to be especially useful when the Bayesian CF framework is applied to RS data acquired in clinical populations (e.g., with a lesioned visual pathway or brain neurodegeneration). In general, we conclude that FWE *beta*-thresholding is a useful complementary approach to the standard VE thresholding for both VFM and RS data.

### Relationship Between Resting State Signals and Functional Architecture

Recent studies have shown that indirect measures of intrinsic neuronal activity, such as spontaneous BOLD fluctuations recorded during RS, can still reflect the organization of the neuroanatomical connectivity that characterizes early visual cortical areas. These studies have allowed the assessment of both fine-grained within- and between- area interactions. This observed spatial specificity in spontaneous BOLD fluctuations can only emerge if these are anchored in the topographically organized architecture of the visual system, as has been shown on multiple occasions (Biswal et al., 1995; Baseler et al., 1999; Azzopardi and Cowey, 2001; Haak et al., 2013b). However, the neuronal and physiological basis of these BOLD patterns is still unclear. Whether spontaneous fMRI activity reflects the consequences of local population spiking activity, sub-threshold neuronal activity (Logothetis et al., 2001; Shi et al., 2017), or metabolic relationships between neurons and astrocytes (e.g., neurovascular coupling) is still a matter of debate (O’Herron et al., 2016; Pang et al., 2017). It is possible that retinotopically organized inter-areal BOLD coupling patterns reflect intrinsic activity in distant cortical areas, sharing similar selectivity in visual field positions which is likely due to “hard wired,” i.e., white matter bundle coupling. Alternatively, these patterns may reflect the footprint of slow fluctuations that traverse the brain like “waves” (Logothetis and Wandell, 2004; Carandini et al., 2015). Recent studies have unified these contrasting views by showing that both global fluctuations, in the form of propagating hemodynamic waves, and transient local coactivations are necessary for setting the spatial structure of hemodynamic functional connectivity (Pisauro et al., 2013; Matsui et al., 2016). Taken together, these studies point to the multiple roles that neuroanatomical, physiological and vascular factors play in shaping spontaneous RS activity in a way that gives rise to visuotopically organized fluctuations in the BOLD signal. The similar visual field position selectivity revealed by RS- and VFM-derived CF maps, suggest a shared neuroanatomical origin.

### Limitations and Future Directions

Here, we qualitatively compared local functional connectivity at different magnetic field strengths obtained in different participants. For a direct comparison, the 3T and 7T derived results should ideally be obtained in the same participants. However, in our view, the differences in the acquisition protocols are minor and were no reason to burden a new cohort of participants to obtain new scans. Nevertheless, while we indeed report a good level of agreement between the CF estimates obtained using the RS and VFM scans, future studies should consider using identical MR parameters for the VFM and RS scans. Moreover, such future studies could investigate the correlations in the temporal and spatial domain in the cortex extending the Bayesian CF model to capture distinct dynamics in functional connectivity, and their relationship to different cognitive and behavioral states, both in health and disease. Furthermore, such studies could also consider the influence of high frequency fluctuations (above 0.1 Hz) in the spontaneous BOLD signal (Chen and Glover, 2015) on CF parameter estimates. Finally, the stimulus-agnostic and eye-movement independent character of the CF analysis invites applying the present approach also to other cortical regions, such as those involved in auditory, somatosensory, or motor processing (Knapen, 2020).

## Conclusion

We have shown that CF modeling is a suitable tool to characterize and quantify the local functional connectivity of visual cortical areas during resting state at 3T. Moreover, the CF modeling at 3T provides qualitatively similar results to those previously observed at 7T, indicating that this lower, yet much more commonly available, field strength would be sufficient for characterizing the brains of patients and individual cases. Finally, we show that our novel Bayesian CF modeling approach provides additional and independent parameters such as uncertainty and effect size that, in principle, can be used to compare the local functional connectivity over different conditions, models and/or groups and assess the statistical significance of the modeling.

## Data Availability Statement

Data will be made available under request. Requests to access these datasets should be directed to the corresponding author: a.invernizzi@umcg.nl.

## Ethics Statement

The studies involving human participants were reviewed and approved by the UMCG. The patients/participants provided their written informed consent to participate in this study.

## Author Contributions

AI, NG, KH, RR, and FC conceptualized and designed the study. AI performed the data analysis and visualization. AI and NG wrote and finalized the manuscript. KH, RR, and FC supervised the study and revised the final draft of the manuscript. All authors read and approved the final manuscript.

## Conflict of Interest

The authors declare that the research was conducted in the absence of any commercial or financial relationships that could be construed as a potential conflict of interest.

## Footnotes

1 https://github.com/Psychtoolbox-3/Psychtoolbox-3/

2 http://www.itk.org

3 http://vistalab.stanford.edu/

4 http://itk.org

## Supplementary Material

Supplementary Figure 1 Visualization of CF maps of non-denoised and denoised RS data for a single subject. From left to right: eccentricity, polar angle and CF size. **(A)** corresponds to VFM derived estimates. It is reported to serve as reference for estimates obtained using RS data. **(B,D)** show CF parameters for each RS run before applying ICA-AROMA denoising procedure. While **(C,E)** show CF estimates for RS1 and RS2 after applying the denoised procedure.

Supplementary Figure 2 Comparison of thresholding approaches on a single subject level in V1 > V2 area using RS1 data. In **(A)** the relation between VE and the beta parameter is presented for all the voxels (orange diamonds) and only for the ones surviving the 95% CI FWE beta-threshold (blue dots). The standard VE threshold is not applied but indicated by a black dotted line. In **(B)** the relation between VE and the uncertainty associated with beta is presented.

Supplementary Figure 3 Connective field size as a function of pRF eccentricity for RS scans. For standard and Bayesian CF models, eccentricity was binned in intervals of 1° and a linear fit was applied. The CF size was initially weighted with variance explained higher than 0.15. Each dot and triangle indicate the mean of the CF size for each bin. While the dashed lines correspond to the 95% bootstrap confidence interval of the linear fit. In **(A)** CF models were applied to RS1 scan while, in **(B)** to RS2 scan.

Supplementary Figure 4 Evaluation of different VE thresholds on ICC. In order to evaluate a viable VE threshold applied on the test-retest analysis, we evaluate the influence of using five different% of strongest activated voxels based on VE (1%, 5%, 10%, 25%, and 50%) on the final ICC (r) across ROIs **(A–E)**. Each participant is represented by a colored lines.

Supplementary Table 1 Correlation between non-denoised and denoised CF maps obtained from RS data at group level. To estimate and compare the level of agreement between not-denoised and denoised CF maps that were obtained from RS1 and RS2 scans by using the standard CF model, we computed the Pearson’s correlations for the eccentricity (*rho*) and the circular correlation for the polar angle (*theta*) parameters. In order to compute the correlation scores, eccentricity and polar angle parameters were estimated at single subject level and then concatenated across all participants.

Supplementary Table 2 Within-subject variability of CF parameter estimates. For standard and Bayesian CF models, we estimated the coefficient of variation to evaluate the within-subject reproducibility of eccentricity and polar angle estimates for both RS scans. The coefficient of variation is reported for each visual area and for each participant.
