# Supplementary material for: Assessing Uncertainty and Reliability of Connective Field Estimations From Resting State fMRI Activity at 3T
Source: Front Neurosci. 2021 Feb 22;15:625309. doi: 10.3389/fnins.2021.625309 (PMC7937930; doi:10.3389/fnins.2021.625309)
Supplement: Supplementary Figure 1 — Visualization of CF maps of non-denoised and denoised RS data for a single subject. From left to right: eccentricity, polar angle and CF size. (A) corresponds to VFM derived estimates. It is reported to serve as reference for estimates obtained using RS data. (B,D) show CF parameters for each RS run before applying ICA-AROMA denoising procedure. While (C,E) show CF estimates for RS1 and RS2 after applying the denoised procedure. [file Data_Sheet_1.pdf]

Supplementary Material

Bayesian connective field model

The following description is adapted from Invernizzi et al. (2020).

Based on the CF definition used in the standard approach (Haak et al. 2013b), a linear spatiotemporal model and a 2D symmetric Gaussian connective field model (2) are used to create a predicted time serie ($p(t)$) which is fitted to the time series $y(t)$of a target location (1) .

$$y(t) = p(t)\beta + \varepsilon \quad (1)$$

$$g(v) = exp - [D(v, v_0)^2 / 2\sigma^2] \quad (2)$$

$$p(t) = \sum_v [a(v,t) * g(v)] \quad (3)$$

Where the predicted fMRI signal $p(t)$ is obtained by the overlap between the CF model $g(v)$ and the neuronal population inputs $a(v,t)$, that are defined as the BOLD time series (converted to percent signal change) for voxels (v) (see eq. 3). In equation 1, β defines the effect size and ϵ is the error term.

The 2D symmetric Gaussian CF model of voxel (v), $g(v)$ is defined based on the shortest three-dimensional distance $D(v, v_0)$ between a voxel (v) and the proposed CF center (v_0) on a triangular mesh representation and σ, which defines the width of the CF. D is computed using Dijkstra's algorithm while σ is constrained to the range $[r_0\ r]$ using a latent variable l_σ(Zeidman et al. 2018). A flat prior is assumed for σ. Therefore, the prior for the latent variable l_σ is defined as a normal distribution $N(0,1)$(see equation 4). As explained in Zeidman et al.(Zeidman et al. 2018), each latent variable is assigned to a prior distribution that represents our beliefs for that CF parameter, before the model fitting.

$$\sigma = (r - r_0) * NCDF(l_\sigma, 0,1) + r_0 \quad (4)$$

Where$r$is the maximum radius and $r_0$is the smallest allowed radius for the CF width - that can be an arbitrarily small non-zero number, which here were set to 10.5° and 0.01°, respectively. $NCDF$indicates

the normal cumulative distribution function.

The MCMC is an iterative sampling approach. During each iteration the parameters for a new CF are set and the fit is compared against the current one. A new location will be selected using the distance to the current position ($d_{current}$). Based on the distance matrix (D), the maximum step (ms) possible in the source region was defined as half the maximal distance from the the current position ($d_{current}$) (5). Latent variable l_s, is randomly drawn from a normal distribution N(0,1) which results in a flat prior for the step size ($step$) between 0 and the maximum step [0 ms] (5, 6). The updated sampling position ($v_{0\ proposal}$) is defined as that position for which the distance to the current position is as close as possible to $step$. If multiple locations are found, only one is drawn randomly.

$$ms = max\ (d_{current})\ /\ 2 \qquad (5)$$

$$step = |ms * NCDF(l_s, 0,1)| \qquad (6)$$

Note that for the first iteration the CF center (v_0) was randomly selected from the source region.

Simultaneous with an updated sample location, an updated width for the CF is calculated. The $l_{\sigma\ proposal}$ is drawn from a gaussian distribution centered around the current value with a width $w_{proposal}$ (7).

$$l_{\sigma\ proposal} = N(l_\sigma, w_{proposal}) \quad (7)$$

The effect size (β) is estimated in parallel to the other CF parameters and constrained to be positive (Zeidman et al. 2018) using the following equation:

$$\beta = exp(l_\beta)\ (8)$$

A latent variable l_β was defined with a prior distribution $N(-2,5)$ and the nextβvalue was controlled by $l_{\beta\ proposal}$(9).

$$l_{\beta\ proposal} = N(l_\beta, w_{proposal})(9)$$

In this study, the initial values of l_σ, $l_\beta$and $w_{proposal}$ were set to 1 , -5 and 2, respectively.

At each iteration of the MCMC, the updated CF parameters (σ, β) were estimated using the following steps. First a predicted fMRI signal $p(t)$ is generated from the source region using eq 2. Note that $g(t)$ was scaled to ensure that the total area under the gaussian, as calculated across the full source region, was equal to one. Second, the error per time point $e_t$between the measured fMRI signal ($y(t)$) and the predicted fMRI signal $p(t)$ was calculated. $e_t$is calculated via subtraction of the predicted signal $p(t)$ from the measured fMRI signal. Then, the log-likelihood L_t associated with e_t was estimated using equation (10). We assumed that e_t follows a standard normal distribution: *N*(0,1). After estimating the mean and standard deviation of ϵ ($\widehat{\mu_\varepsilon}$ and $\widehat{\sigma_\varepsilon}$) we calculated the maximum likelihood estimates (MLE, eq. 11).

$$L_t = log(N(-|e_t|, \widehat{\mu_\epsilon}, \widehat{\sigma_\epsilon}) \qquad (10)$$

$$MLE_B = \sum_t L_t + log(N(l_\sigma, 0,1)) + log(N(l_\beta, -2,5)) \qquad (11)$$

At this point, MLE of the proposal iteration is compared to the last accepted (current) sample based on an Accepted ratio score Ar (12).

$$Ar = exp\,(MLE_{t\,proposal} - MLE_{t\,current}) \; (12)$$

$Ar$was compared to a pseudo-random acceptance score defined as a normal distribution $N(0,1)$ and only if the Ar was higher, the respective latent variables were updated. Based on the accepted l_σ, l_s and l_β values, a new CF was defined and a new MCMC iteration took place.

Supplementary Figures

To check the possible influence of the ICA-AROMA denoised procedure, the same quantification analysis was computed on non-denoised RS data. Similar maps (Figure 1S) and correlation values (Table 1S) were observed indicating that the ICA-AROMA denoised procedure on RS-fMRI data did not influence the final CF outcomes.

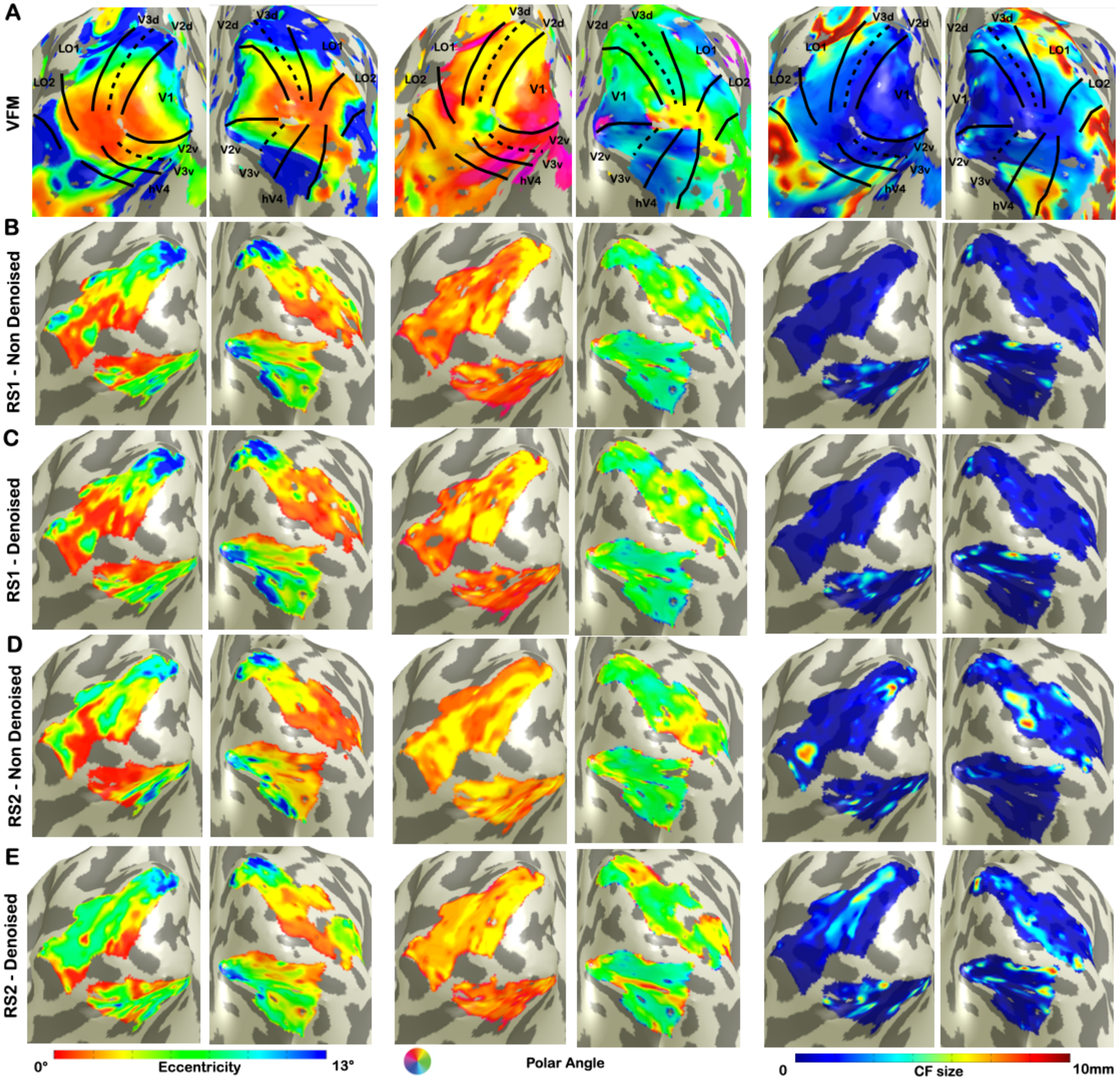

Figure 1S. Visualization of CF maps of non-denoised and denoised RS data for a single subject. From left to right: eccentricity, polar angle and CF size. Panel A corresponds to VFM derived estimates. It is reported to serve as reference for estimates obtained using RS data. Panels B and D show CF parameters for each RS run before applying ICA-AROMA denoising procedure. While panels C and E show CF estimates for RS1 and RS2 after applying the denoised procedure.

**Eccentricity**				
ROIs	***RS1***		***RS2***	
	Non Denoised versus Denoised		***Non Denoised versus Denoised***	
	R	*p-value*	*R*	*p-value*
V1 --> V2	0.292	p<0.001	0.0763	0.0247
V1 --> V3	0.1353	0.0003	0.1059	0.0366
V1 -> hV4	0.2124	0.0251	0.1923	0.1068
V1 -> LO1	0.1808	0.0472	-0.037	0.0912
V1 -> LO2	0.0787	0.3095	0.0077	0.5983
**Polar Angle**				
ROIs	***RS1***		***RS2***	
	Non Denoised versus Denoised		***Non Denoised versus Denoised***	
	R	*p-value*	*R*	*p-value*
V1 --> V2	0.362	p<0.001	0.2609	p<0.001
V1 --> V3	0.2451	p<0.001	0.3094	p<0.001
V1 -> hV4	0.3487	p<0.001	0.0862	0.1191
V1 -> LO1	0.3017	p<0.001	-0.041	0.6232
V1 -> LO2	0.2959	0.008	0.0048	0.5649

Table 1S. Correlation between non-denoised and denoised CF maps obtained from RS data at group level. To estimate and compare the level of agreement between not-denoised and denoised CF maps that were obtained from RS1 and RS2 scans by using the standard CF model, we computed the Pearson's correlations for the eccentricity (*rho*) and the circular correlation for the polar angle (*theta*) parameters. In order to compute the correlation scores, eccentricity and polar angle parameters were estimated at single subject level and then concatenated across all participants.

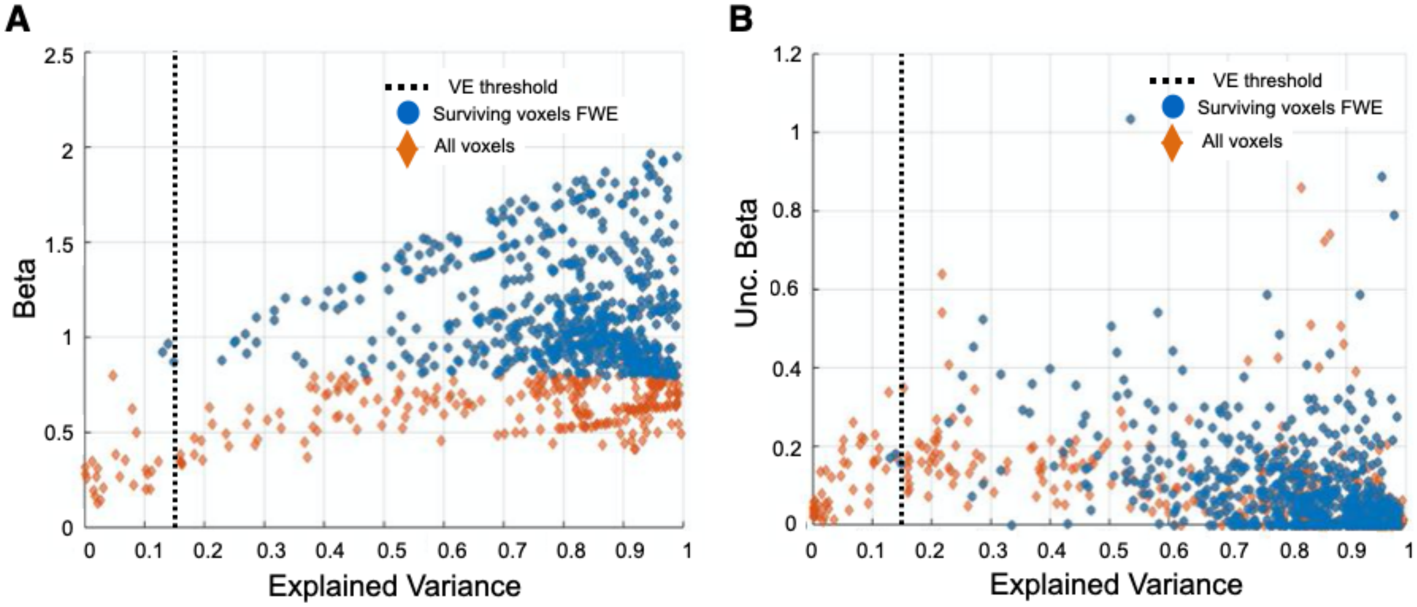

Figure 2S. Comparison of thresholding approaches on a single subject level in V1>V2 area using RS1 data.
In Panel **A**, the relation between VE and the beta parameter is presented for all the voxels (orange diamonds) and only for the ones surviving the 95% CI FWE beta-threshold (blue dots). The standard VE threshold is not applied but indicated by a black dotted line. In Panel **B**, the relation between VE and the uncertainty associated with beta is presented.

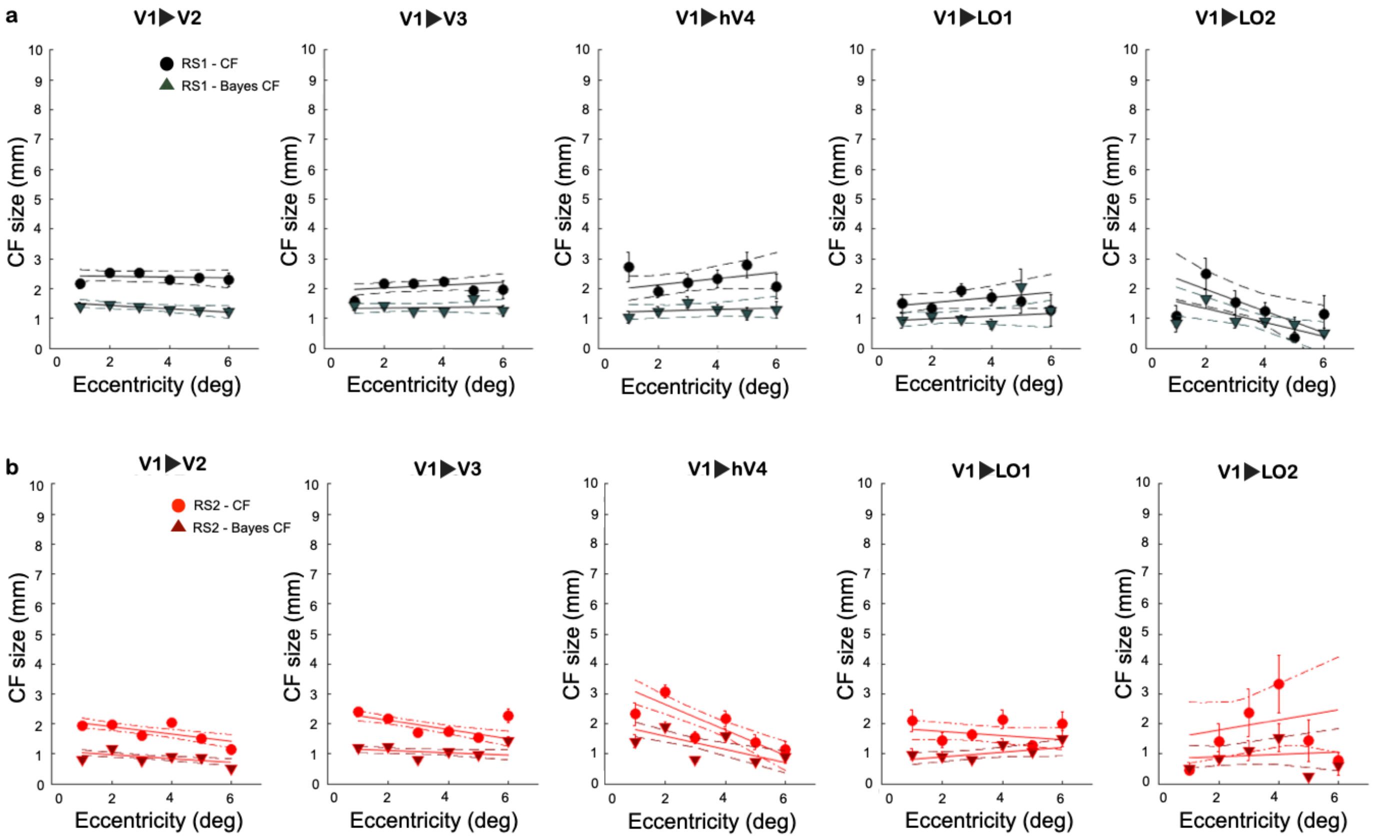

Figure 3S. Connective field size as a function of pRF eccentricity for RS scans. For standard and Bayesian CF models, eccentricity was binned in intervals of 1 deg and a linear fit was applied. The CF size was initially weighted with variance explained higher than 0.15. Each dot and triangle indicate the mean of the CF size for each bin. While the dashed lines correspond to the 95% bootstrap confidence interval of the linear fit. In Panel **A**, CF models were applied to RS1 scan while, in panel **B** to RS2 scan.

Eccentricity					Polar Angle				
V1 > V2	RS1		RS2		*V1 > V2*	RS1		RS2	
	Standard CF	*MCMC CF*	*Standard CF*	*MCMC CF*		*Standard CF*	*MCMC CF*	*Standard CF*	*MCMC CF*
sub1	0.6041	0.6168	0.5982	0.6087	*sub1*	0.5967	0.5847	0.4975	0.4835
sub2	0.4289	0.3591	0.3788	0.3831	*sub2*	0.7881	0.7816	0.6632	0.585
sub3	0.6059	0.5982	0.6099	0.5879	*sub3*	0.5281	0.5111	0.4904	0.4921
sub4	0.4592	0.434	0.5008	0.4773	*sub4*	0.6768	0.586	0.6838	0.6933
sub5	0.5061	0.498	0.5415	0.5343	*sub5*	0.6582	0.6604	0.6482	0.6533
sub6	0.5247	0.5255	0.6121	0.6055	*sub6*	0.366	0.3461	0.4629	0.4349
sub7	0.5014	0.4718	0.5099	0.5425	*sub7*	0.2371	0.2598	0.6426	0.6845
sub8	0.7656	0.7639	0.6928	0.67	*sub8*	0.7514	0.7573	0.6609	0.6571
sub9	0.516	0.5207	0.4651	0.4561	*sub9*	0.7687	0.8129	0.6615	0.6946
sub10	0.8177	0.7275	0.7449	0.7424	*sub10*	0.5176	0.4875	0.4887	0.5002
sub11	0.4719	0.455	0.4962	0.4879	*sub11*	0.4174	0.4228	0.4123	0.466
sub12	0.6945	0.6986	0.6202	0.6218	*sub12*	0.8659	0.8434	0.7764	0.7758
V1 > V3	*Standard CF*	*MCMC CF*	*Standard CF*	*MCMC CF*	***V1 > V3***	*Standard CF*	*MCMC CF*	*Standard CF*	*MCMC CF*
sub1	0.4603	0.4711	0.5611	0.5309	*sub1*	0.7438	0.751	0.5269	0.5182
sub2	0.5583	0.4778	0.3524	0.354	*sub2*	0.9487	1.0093	0.5867	0.571
sub3	0.4518	0.4575	0.4717	0.4765	*sub3*	0.747	0.7454	0.6618	0.6952
sub4	0.4397	0.4053	0.4773	0.4702	*sub4*	0.7172	0.6134	0.8307	0.8071
sub5	0.4741	0.4749	0.4874	0.4885	*sub5*	0.7118	0.7204	0.6634	0.6146
sub6	0.6169	0.6376	0.6873	0.6626	*sub6*	0.2893	0.2643	0.6059	0.6099
sub7	0.4977	0.4137	0.6137	0.6241	*sub7*	0.2491	0.282	0.674	0.7467
sub8	0.702	0.6898	0.5725	0.5604	*sub8*	0.6118	0.6089	0.4903	0.5178
sub9	0.4956	0.4956	0.4392	0.412	*sub9*	0.7956	0.8247	0.7893	0.8048
sub10	0.7669	0.7155	0.7238	0.7511	*sub10*	1.0817	0.9427	0.7521	0.8018
sub11	0.3979	0.416	0.4218	0.4212	*sub11*	0.8994	0.8481	0.7369	0.7225
sub12	0.5977	0.5834	0.5667	0.5789	*sub12*	0.884	0.881	0.5676	0.5641
V1 > hV4	*Standard CF*	*MCMC CF*	*Standard CF*	*MCMC CF*	***V1 > hV4***	*Standard CF*	*MCMC CF*	*Standard CF*	*MCMC CF*
sub1	0.6803	0.5514	0.6843	0.6709	*sub1*	0.7339	0.5723	0.4696	0.4547
sub2	0.7155	0.3835	0.4345	0.4638	*sub2*	0.3995	0.4733	0.6886	0.6467
sub3	0.5993	0.5699	0.4046	0.4228	*sub3*	0.3161	0.3195	0.4269	0.5167
sub4	0.4081	0.3993	0.4535	0.4314	*sub4*	0.5725	0.5617	0.6375	0.6698
sub5	0.6453	0.6517	0.361	0.3569	*sub5*	0.5036	0.5169	0.3745	0.2899
sub6	0.5209	0.4817	0.9185	0.9595	*sub6*	0.1608	0.1561	0.7456	0.7732
sub7	0.5219	0.4745	0.7038	0.7461	*sub7*	0.1106	0.0835	0.2249	0.228
sub8	0.5915	0.5477	0.6996	0.6797	*sub8*	0.4678	0.4568	0.429	0.512
sub9	0.488	0.4699	0.5545	0.3309	*sub9*	0.7568	0.7553	0.8493	0.9329
sub10	0.8293	0.338	0.3744	0.4632	*sub10*	0.8133	0.3699	0.3988	0.4486
sub11	0.5025	0.3745	0.4939	0.4956	*sub11*	0.4064	0.1668	0.3861	0.3564
sub12	0.8573	0.7866	0.9118	0.8963	*sub12*	1.1269	1.0395	0.4766	0.466
V1 > LO1	*Standard CF*	*MCMC CF*	*Standard CF*	*MCMC CF*	***V1 > LO1***	*Standard CF*	*MCMC CF*	*Standard CF*	*MCMC CF*
sub1	0.2957	0.3212	0.4511	0.3655	*sub1*	0.7714	1.0436	1.0286	0.997
sub2	0.6151	0.6417	0.1581	0.167	*sub2*	1.1331	0.6344	0.2463	0.2573
sub3	0.4004	0.3685	0.3895	0.3744	*sub3*	1.1454	1.337	0.9311	0.7291
sub4	0.4696	0.4613	0.5243	0.5156	*sub4*	0.997	0.9376	1.4503	1.464
sub5	0.5664	0.5902	0.3486	0.3216	*sub5*	0.8792	0.8429	0.5201	0.4817
sub6	0.5093	0.3655	0.9833	0.9179	*sub6*	0.1371	0.0719	1.2361	1.2842
sub7	0.5554	0.5631	0.6132	0.5848	*sub7*	0.4587	0.4373	0.7042	0.7373
sub8	1.473	1.5283	0.7314	0.5999	*sub8*	0.6043	0.572	0.569	0.4727
sub9	0.6907	0.6924	0.6877	0.9959	*sub9*	0.616	0.6355	0.1301	0.166
sub10	0.7547	0.3716	0.8081	0.8268	*sub10*	0.4037	0.4244	0.5949	0.5881
sub11	0.6004	0.5245	0.4417	0.421	*sub11*	0.7894	0.7604	1.0417	1.0394
sub12	0.3531	0.3709	0.8008	0.8169	*sub12*	0.5541	0.7711	0.3569	0.391
V1 > LO2	*Standard CF*	*MCMC CF*	*Standard CF*	*MCMC CF*	***V1 > LO2***	*Standard CF*	*MCMC CF*	*Standard CF*	*MCMC CF*
sub1	0.0979	0.1385	0.5747	0.6199	*sub1*	0.1007	0.1215	0.8105	0.7892
sub2	0.5392	0.5365	0.2286	0.2218	*sub2*	0.4958	0.3849	0.4824	0.4519
sub3	0.5016	0.5164	0.304	0.2323	*sub3*	1.0287	1.0428	0.5166	0.3704
sub4	0.4251	0.4276	0.4635	0.4391	*sub4*	0.5652	0.5514	1.1707	1.2203
sub5	0.453	0.4733	0.275	0.2578	*sub5*	0.6998	0.7054	0.2904	0.4427
sub6	0.4474	0.2758	0.431	0.3575	*sub6*	0.0767	0.0513	0.2398	0.3694
sub7	0.6104	0.6019	0.6052	0.6216	*sub7*	0.6528	0.7378	0.6285	0.6526
sub8	0.3177	0.2784	0.5604	0.4616	*sub8*	0.0839	0.0779	0.4074	0.3213
sub9	0.5182	0.4935	0.2362	0.2362	*sub9*	0.5656	0.6322	0.1387	0.1387
sub10	0.1187	0.1199	0.3165	0.3103	*sub10*	0.0091	0.009	0.7048	0.8607
sub11	0.9313	0.6467	0.2447	0.4321	*sub11*	0.4989	0.5146	0.2218	0.3629
sub12	0.059	0.2433	0.001	0.0021	*sub12*	0.1179	1.0552	0.002	0.0021

Table 2S. Within-subject variability of CF parameter estimates. For standard and Bayesian CF models, we estimated the coefficient of variation to evaluate the within-subject reproducibility of eccentricity and polar angle estimates for both RS scans. The coefficient of variation is reported for each visual area and for each participant.

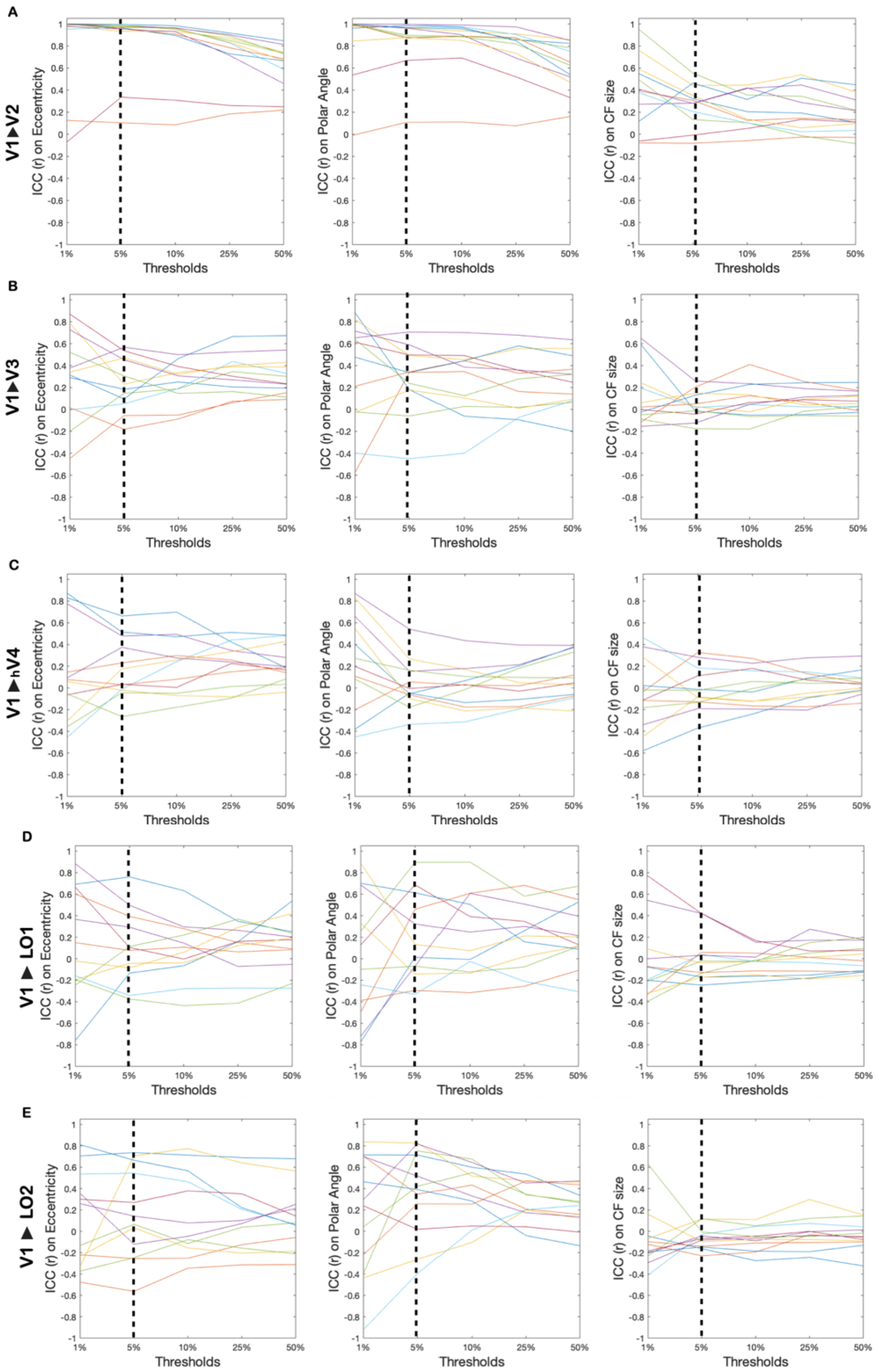

Figure 4S. Evaluation of different VE thresholds on ICC. In order to evaluate a viable VE threshold applied on the test-retest analysis, we evaluate the influence of using five different % of strongest activated voxels based on VE (1%, 5%, 10%, 25% and 50%) on the final ICC (r) across ROIs (Panels A, B, C, D and E). Each participant is represented by a colored lines.
